# “Good Food Causes Good Effects and Bad Food Causes Bad Effects”: Awareness, Meanings and Perceptions of Malnutrition among Pakistani Adolescents Living in Slums through Photodiaries and Interviews

**DOI:** 10.3390/nu15010033

**Published:** 2022-12-21

**Authors:** Sara Estecha-Querol, Syeda Kisa Zehra Zaidi, Lena Al-Khudairy, Paramjit Gill, Romaina Iqbal

**Affiliations:** 1 Warwick Centre for Global Health, Warwick Medical School, University of Warwick, Coventry CV4 7AL, UK; 2 Division of Health Sciences, Warwick Medical School, University of Warwick, Coventry CV4 7AL, UK; 3 Department of Community Health Sciences, Aga Khan University, Karachi 74800, Pakistan

**Keywords:** adolescent, malnutrition, slum, Pakistan, qualitative research, photodiary, thematic analysis

## Abstract

Around 30% of the urban population of Southern Asia lives in a slum setting where basic necessities such as sanitation, education, employment, infrastructure are lacking, and people are more exposed to health problems. Children living in slums are at high risk of malnutrition. However, there is limited knowledge on adolescents living in slums. We explored awareness and meanings of malnutrition (under and over-nutrition) as well as examining malnutrition risk factors among adolescents living in a slum. A qualitative approach was undertaken using photodiaries and semi-structured interviews with 14 adolescents (13–16 years old) living in a slum in Karachi (Pakistan). An inductive strategy was used moving from open-ended data to patterns using reflexive thematic analysis. We identified widespread malnutrition awareness amongst these adolescents. Food consistently underpinned participants’ narratives and photodiaries, which was reflected in how they made sense of malnutrition: undernutrition was understood as lack of food, while overnutrition as excess of food. This study identified malnutrition drivers: sanitation, exercise, families, peers, wellbeing, gender, nutritional knowledge, media, and most importantly, food. People’s responsibility to eat healthily was highlighted by the participants, implying that people are to be blamed for their poor lifestyle choices. Following this responsibility discourse, most participants contemplated merely individually focused health interventions in order to overcome the problem of malnutrition in their area. It is necessary to study slum food environments better to implement effective nutrition programs and interventions to reduce malnutrition in slum settings.

## 1. Introduction

Malnutrition is a highly prevalent and persistent problem in low-income and middle-income countries (LMICs), and its consequences come at a high human and economic cost [1]. The World Health Organization (WHO) defines malnutrition as the deficiency, excess or imbalance of energy and/or nutrients. The term malnutrition covers several conditions across the life-course such as overnutrition (overweight/obesity), micronutrient deficiencies, and undernutrition (underweight, stunting and wasting) [2]. Every country in the world is affected by one or more forms of malnutrition, making this health issue one of the greatest global challenges [3]. In South Asia, while the prevalence of undernutrition remains highly concerning, the prevalence of overnutrition is currently rising [4]. Health and nutritional surveys in South Asia have mainly focused on malnutrition among children under five years old and women of reproductive age [5]. Although adolescence provides a window of opportunity for improving nutrition [6], the burden of malnutrition among adolescents remains unattended and insufficiently investigated in LMICs [7], particularity in slum settings [8,9].

The rapid urbanisation of LMICs in the past 50 years has driven the expansion of slums—also called deprived urban neighbourhoods—that are now home to approximately one billion people [10,11]. The UN defines a slum as an informal urban settlement that lacks one or more of the following conditions: access to clean water, access to improved sanitation, sufficient living area, durable housing, and security of tenure [12]. This urban growth sets an unprecedented global challenge as slum dwellers have limited access to education and social services and are more exposed to health problems such as child mortality, infectious diseases and undernutrition [11,13]. Recent studies in South Asia estimated the magnitude of malnutrition among adolescents living in slums: stunting prevalence was 9% [14], 22% [15] and 43% [16]; thinness was 24% [14], 60% [17] and 61% [16]; overweight was 11% [14]; and anaemia was 90% [16]. Factors associated with adolescent nutritional status were sex (boys were more stunted and thin than girls) [14], parental education (poor parental education was associated with stunting, thinness and overweight) [14], age (younger adolescents were more thin than older adolescents) [17] and socioeconomic status (low socioeconomic status was associated with thinness) [17].

Adolescent malnutrition has been traditionally investigated using quantitative approaches [8] despite the efforts of international agencies in highlighting the importance of using qualitative approaches with adolescent populations [18,19]. Qualitative methods have been employed to investigate health care providers’ and caregivers’ awareness and perspectives on childhood malnutrition in LMIC [20,21,22,23] and in slum settings [24,25,26]. However, qualitative studies exploring adolescent malnutrition are lacking in these particular contexts. Adolescents’ perspectives on malnutrition could be valuable to gain a better understanding of the problem, challenge the purely quantitative definition of adolescent malnutrition and potentially reveal a new conceptualisation of malnutrition, including “how it feels” and “how it is understood”. Therefore, the aims of this study were to (i) explore awareness and perspectives of malnutrition, and (ii) identify determinants of malnutrition among adolescents living in a slum in Karachi (Pakistan) using a qualitative method.

## 2. Materials and Methods

### 2.1. Qualitative Approach and Research Paradigm

This qualitative study used pragmatic theoretical assumptions to understand malnutrition in a given setting. Following pragmatism worldview, we made decisions on how to best answer our research questions sensibly considering the context (slum in an LMIC), current world situation (COVID-19 pandemic) and research practicalities (time and resources available). This research study draws on an interpretive approach and is exploratory in nature, using an inductive strategy that moves from open-ended data to patterns. A thick narrative of the data extracts is presented and discussed, moving from a descriptive to an interpretative level and bringing in the existing literature [27,28]. Established frameworks on adolescent nutritional status [6,29,30,31] were used as a lens through which to develop the research aims, design the methods, and interpret the data rather than hypothesis or (quantitative) theory testing [32].

### 2.2. Setting

This research study took place in a slum in the city of Karachi (Pakistan) which is a centrally located, well-established neighbourhood in Karachi East district [33]. Approximately 0.1 million people live there, and the population is mostly working in blue-collar jobs. There are several ethnic groups in this slum and the religion is mixed, having one of the largest proportions of Christians in the city (40%). Structures are permanent and multistorey, with high levels of new construction underway. Residents have variable access to basic services and sanitation [33].

### 2.3. Recruitment and Sample Characteristics

The fieldwork took place from March to August 2021. Both door-to-door and school-based recruitments were performed in order to maximise the chances to recruit participants during COVID-19. In total, 14 adolescents (13–16 years) living in a slum in Karachi were included (Table 1). All participants were attending private schools. This study, therefore, involved convenience and purposive sampling where the selected sample was accessible and could potentially contribute with rich data [34].

Braun and Clarke have recently discussed how the claim of achieving “data saturation” to determine sample size is inherently problematic in qualitative research, especially in more interpretative forms of qualitative research [35]. Considering this study’s approach and its method for data analysis, the sample size was not determined by seeking data saturation but rather by making pragmatic decisions around sampling [35]. Hence, the optimal sample size for this study was determined to be 14 participants by taking into account the research question, the purpose of the project, the methods of data collection, the homogeneity of the sample, the COVID-19 pandemic, the expected depth of data generated from adolescent participants, and the time and resources available.

### 2.4. Data Collection Methods and Instruments

Engaging, fun, interactive and creative data collection methods are most appropriate for conducting research with young children and adolescents [18]. Considering participants’ age and context (i.e., literacy level, cultural sensitivities, COVID-19, and limited access to the internet or mobile devices), a qualitative method including images or visual data such as photodiaries was perceived as suitable by the research team.

The photodiary method was initially developed to investigate Muslim and African Caribbean teenagers’ experiences on parenting in Hirst’s study in 2003 [36]. Participants take pictures over a determined period of time following prompts or instructions given by the researcher as well as writing a diary recording their thoughts, feelings or experiences about the given research topic. The researcher initiates and facilitates the assignment [34] but the participant generates the material and therefore chooses what to capture and record in the photodiary [37].

Informed consent was obtained from adolescents and one parent/guardian. High illiteracy levels were expected among parents of potential participants. To ensure compliance with the highest ethical standards, as well as participants’ and parents’ understanding of the aims and process of the project, a phone-based verbal consent and written consent were taken. Urdu consent forms and lay language information sheet were provided. The research assistant (S.K.Z.Z.) explained the project and ensured that both the participant and parents understood it. In addition, a community health worker acted as a witness and was present.

After taking consent, participants received a notebook, colour pens, glue, scissors, a Polaroid camera (INSTAX^®^ Mini 11), and detailed photodiary instructions to complete the research project. Polaroid cameras contain a self-developing film to create a print shortly after taking the picture without requiring any additional electronic devices or the internet. The photodiary instructions (Supplementary Material S1) were informed by relevant frameworks on adolescent nutritional status [6,29,30,31]. The instructions were designed to prompt reflection on the meaning of malnutrition, and how malnutrition and its associated factors affect participants and their community. Participants were expected to take photos of anything they perceived to be related with malnutrition (undernutrition, micronutrient deficiencies and overnutrition) and paste these pictures into the diary along with a written note explaining the content of the image. However, we recognise that snapping these reflections could have been challenging for participants due to the (abstract) nature of the topic and the newness of the method. In the same way, a study using photodiaries to promote critical language awareness among Spanish language learners asked “what is Spanish-speaking culture?” [38]. Since the ‘task’ was not easy to comprehend and perform, we implemented strategies to mitigate potential misunderstandings and technical problems with the Polaroid camera. S.K.Z.Z. carried out regular follow-up calls and visits to the schools or households to not only offer practical support but also to aid memory and motivation.

Participants were given two weeks to complete their photodiary. After this time, photodiaries were collected, scanned and returned to the participants. Finally, face to face semi-structured interviews were conducted with the participants by S.K.Z.Z. Informed by relevant frameworks on adolescent nutritional status [6,29,30,31], a topic guide was prepared for this study (Supplementary Material S2). The topic guide had two distinct parts: (i) questions about the content of the photodiary (this part was tailored for each participant, see an example in Supplementary Material S2), and (ii) questions on the concept and perceptions of malnutrition and how this affected the participant and their community. S.K.Z.Z. conducted the interviews in Urdu which lasted from 15 to 37 min. Previous consent (verbal) was given by the participant before starting the audio recording. S.K.Z.Z. transcribed the photodiaries and audios verbatim in Urdu, and then translated them from Urdu to English (semantic translation). Quotes from the photodiaries are in Urdu unless indicated otherwise.

### 2.5. Data Analysis

Reflexive thematic analysis (TA) following Braun & Clarke’s latest practical guides [32,39] was used as a method for data analysis in this study. Reflexive TA was used for two main reasons: (i) its suitability to explore people’s experiences, perspectives, and understandings as well as explore the influencing factors that shape a particular phenomenon; and (ii) its flexibility to be applied in different datasets and within a huge range of theoretical frameworks [32]. The development of codes and themes was directed by the content of the data (more inductive) and reflected the explicit or manifest meanings of the data (more semantic) [40].

Firstly, the first author, S.E.-Q., became familiarised with the data by actively reading the field notes, transcribing interviews and transcribing photodiaries while noting initial thoughts about coding and items of potential interest using annotations and memos in Nvivo 12. Notes were taken from individual data items (e.g., an interview or a photodiary) and in relation to the entire dataset. Data were coded by S.E.-Q. using Nvivo 12. After re-reading all codes, some were renamed, some broken into more codes and others transferred to another code due to their similarity. In total, 206 codes were identified. Next, S.E.-Q. generated seven initial themes. At this phase, a visual thematic map was built to sort the codes into themes, and a flow chart was created to represent the relationship between themes. The central organising concept and its boundaries were written down as a ‘story’ or ‘abstract’ for each theme. Further reviewing and defining the themes resulted in three themes. The central idea and the boundaries of these new three themes were written. All the phases, especially these themes, were shared and reviewed with L.A.-K. to construct a richer reading of the data and, consequently, themes were refined.

Checklists for reporting reflexive TA were followed [28,40]. Lincoln and Guba (1985) also established strategies to guarantee quality [41]. The following techniques were used in this study: (i) prolonged engagement in the field with participants; (ii) audit trail by transparently describing the decisions made and steps taken during the research process; (iii) “thick description” of the research process; (iv) methodological triangulation by gathering data with different collection methods (participant’s diary, interviews, research assistant’s diary (S.K.Z.Z.) and researcher’s diary (S.E.-Q.)); (v) triangulation by interpreting and reviewing sessions with the research team; and (vi) reflexive diary from both S.K.Z.Z. and S.E.-Q. on roles, performance, preconceptions and values (see a critical reflection in Supplementary Material S3).

### 2.6. Ethics

This research complied fully with the ethical practice guidelines laid out by three institutions: the University of Warwick (reference number BSREC 74/19-20 AM02), the Aga Khan University (reference number 2021-3717-16861) and the National Bioethics Committee Pakistan (reference number 4-87/NBC-630/21/1578).

## 3. Results

The analysis produced three final themes. The first theme described the role of (good or bad) food in health and nutrition status. The second theme focused on the participants’ social context influences concerning their dietary intake and food choices. The third theme explored participants’ preconceptions of being healthy.

### 3.1. Theme 1: Malnutrition Is All about (Good or Bad) Food

**Figure 1:**
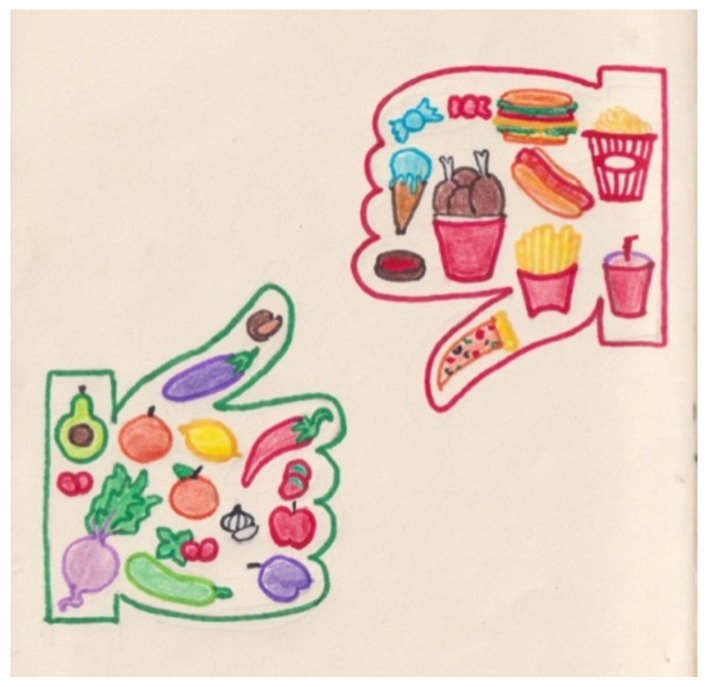
Rimsha’s photodiary: representation of the good and the bad food.

Food, specifically, what participants understood by good or bad food, recurred throughout the dataset. In photodiaries, there was a common intention to capture a variety of food items and classify them into two distinct categories, as Sakina’s title diary illustrates: “merits and demerits of food” [written in English]. Generally, participants identified vegetables and fruits as “healthy” or “good”, and a diet based on those items would be “good for health”, “balanced”, “right”, or “proper”. However, junk food, sugar, fried food, soft drinks, and snacks were classified as “unhealthy”, “bad”, or “harmful”, and individuals consuming those would have a “poor quality diet”, which would be “bad for health”. The ‘good’ and the ‘bad’ continuously emerged from the interviews and photodiaries (Figure 1). Food and beverages were viewed as either good or bad for health, with no middle ground with the exception of meat, bread, potato and rice. There was some controversy and confusion about the healthiness of these products amongst participants. Interestingly, the first pages usually showed good food items and their “benefits”, while the last pages contained bad items (including alcoholic drinks, tobacco and smokeless tobacco) and their “side” or “adverse effects”. Participants’ (almost exclusive) focus on snapping pictures of good or bad food suggests that their understanding of malnutrition was in some way limited to the relationship between both. Rimsha’s interview provides an example of this:

*Interviewer: the pictures you have pasted [in your diary] are mostly food items. So, how food items are related to undernutrition and overnutrition?*

*Rimsha: if we eat good food there will be a good effect on our body and if we eat bad food there will be bad effect on our body. That is, good food causes good effect and bad food causes bad effects. It is said like that if we eat fruits, it will have a good effect. And whatever we eat good or bad, it goes into our stomach, so there must be an effect. So whatever we eat good or bad our body will get an effect of that, so good food will produce good effects and bad food will produce bad effects.*

**Figure 2:**
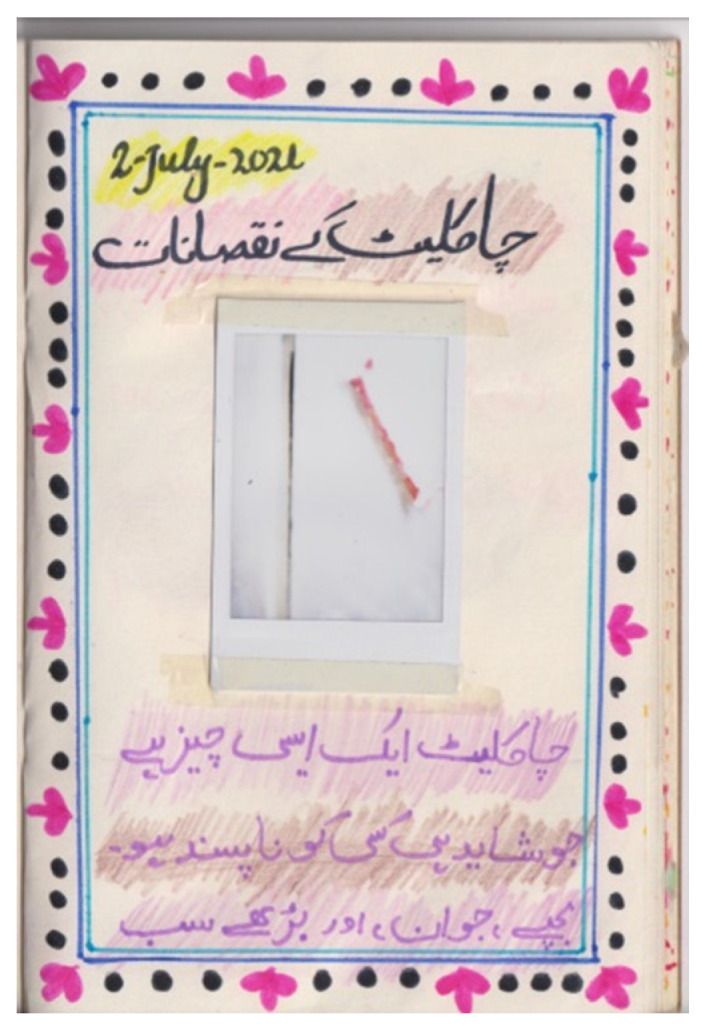
Ashi’s photodiary: “adverse effects of chocolate. Chocolate is something that hardly anyone dislikes. Kids, adults and elderly all like [continues on another page, not shown] to eat chocolate. Although it is delicious in taste, it has adverse effects too”.

Participants photographed and wrote about the most available and consumed foods and beverages. Qirat mentioned that she had to rely on her brothers to bring her the food she wanted to capture, but by the time they brought the desired item, she had already completed her photodiary. Jannat photographed a plate containing six onions and wrote in her diary: “it [onion] is an important vegetable that is consumed in our daily life. Onions are used in Subcontinent, Pakistan and India, and all over the world”. Participants displayed pictures of their favourite food items, acknowledging either the good or bad effects on health. Ashi wrote in her diary a poem about the mango—also known as “the king of fruits” among the participants—which she copied from YouTube. On another page of her photodiary, she pasted a picture of a chocolate bar, indicating its general likeability without forgetting its unhealthiness (Figure 2).

Participants’ efforts to make colourful, well-designed, thorough diaries is apparent, combining photos, drawings, and clippings from magazines as well as writing a few sentences about the content of the illustrations. Mahi drew a dolphin on the cover of the diary, and Mary stuck some post-it page markers on the cover with words such as “healthy”, “sweet”, “food”, “unhealthy”, and “fruit” [written in English]. Sakina wrote on the first page: “it’s my Diary. My Diary is my most important subject. Thanks ☺” [written in English]. Many participants expressed that making the diary was “nice”, “a lot of fun”, and “very enjoyable”. Nonetheless, Sakina commented that although she enjoyed it, she was worried about “making any mistakes”. This suggests that some participants may have seen the photodiary as ‘homework’ since this project was embedded in a school context. Hence, their concern about making mistakes could be stemmed both from the belief of a possible evaluation like a ‘school task’ and the fulfilment of researchers’ expectations. Even though it was a big ‘task’, participants appreciated the opportunity to make and learn something new:

*Ali: I was amused while colouring because I am doing this colouring activity after a long… I never had the chance or even thought to do it. I really liked that you have come [to the school] and I have worked with you on this project and I enjoyed it. I have gained more knowledge, I was not aware of so much information. I mean, I had some knowledge but it increases when a person does it by themselves.*

Making this diary was recognised as a learning experience where what food is healthy or unhealthy was the main—and possibly only—takeaway: “from this [diary making experience] I learned that the food which is good for us should be taken and what is not good should be avoided” (Laraib). Moreover, there was a sense of curiosity for nutrition-related knowledge and reflection amongst the participants regarding the whole experience. For instance, Mahi commented that she will find out more information about the food that she eats from now on. Similarly, Sulman stated that he feels confident about understanding “these nutrition things”, but if there is something that he does not know, he will make sure to “practise and understand it”. These are vivid examples of how the photodiary enhanced their ability to reflect on their dietary choices and empowered them to keep learning.

**Figure 3:**
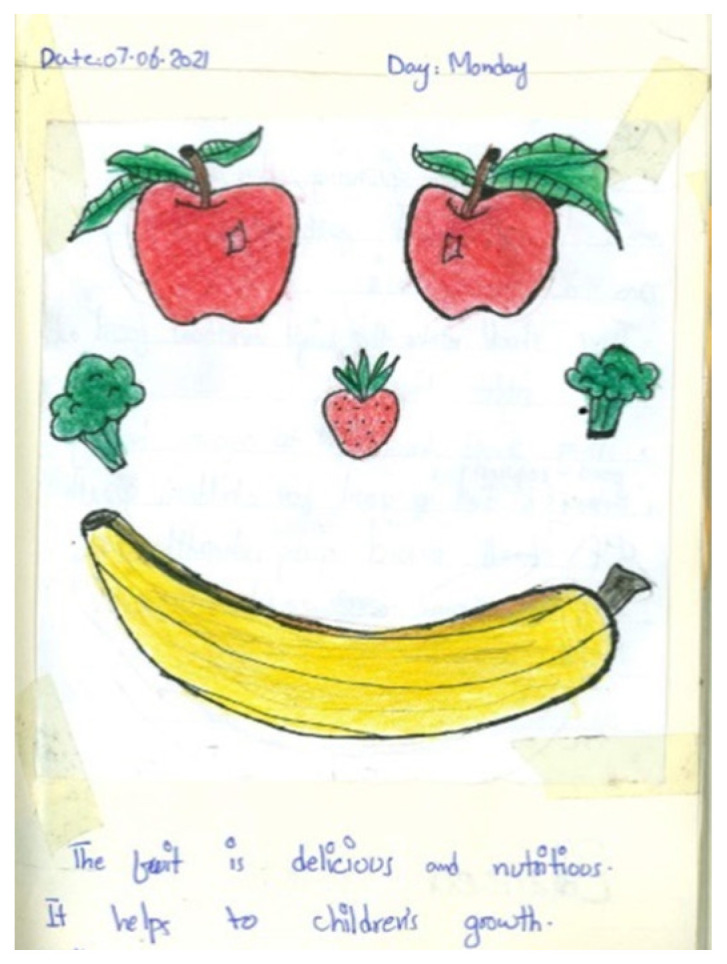
Jon’s photodiary: “the fruit is delicious and nutritious. It helps to children’s growth”.

There was an awareness that nutritious food was essential to their growth and development (Figure 3). Fruits and vegetables were the predilect energy givers amongst the participants. Statements such as “when we eat [fruit], it dissolves inside us and the energy comes into us. Our body grows, our height grows. And more energy also comes into us. As much as we eat fruits, these are so good for health” (Sunny) suggest that growth is as a recursive process that takes places not only on the outside—weight and height increase—but also on the inside. Sunny’s use of the words “energy comes into us” shows how the ‘power of the fruit’ enters the body to subsequently make it grow from the inside to the outside. Sunny viewed ‘having energy inside’ as something desired and visible: “the more we take homemade meal, the more we will show that there is so much strength inside us. Others will say, ‘look at yourself, how much strength is inside you’”. Similar patterns were evident in the accounts of other participants when they described that unhealthy food, smoking and alcoholic drinks bring diseases and germs into the body. Perhaps this reflects an emphasis from their community and household environment to eat healthy food to grow and be in good health.

Food consistently underpinned the participants’ narratives and photodiaries which was reflected in how they made sense of malnutrition. However, there was some confusion when they were asked about the meaning of undernutrition (*ghizayat ki kami*), overnutrition (*ghizayat ki ziyadti*), and vitamin and mineral deficiencies (*haya’teen or madi’niyat ki kami*). A few participants declared not knowing the answer, noted the great difficulty of the questions, or simply went silent. In the Urdu language, *ghizayat ki kami* can be literally translated as deficient in nutrients and *ghizayat ki ziyadti* as an excess of nutrients. Jannat’s description of overnutrition illustrates her sense-making: “overnutrition… when eat properly, it will be fine… not having in excess of things… disadvantage of some things. disadvantage…”. All these responses probably suggest unfamiliarity and/or unawareness with malnutrition words.

Overnutrition was defined as an “excess of food” that appeared to impact people’s bodies and health. There was a general acknowledgement that overnutrition “has a harmful effect on the body” (Mary), and “our body will become fat” (David) if unhealthy items were consumed. The negative impact of eating food in excess was not only limited to these “bad” items; for instance, Sulman and Rimsha commented that eating banana or mango in excess might be detrimental to health. Some participants mentioned that overnutrition was not only related to unhealthy food and beverages but also to physical activity. Physical inactivity was understood to be both the cause and the consequence of obesity. One participant (Sunny) recognised that losing weight was such a challenging and lengthy process. As opposed to overnutrition, many participants described undernutrition as a lack of (nutritious) food: “Not eating… not eating good food… eating fast food, oily food, street food, and not having vegetables, not eating fruits, not drinking milk, not drinking juice” (Rimsha). Undernutrition also appeared to have bodily connotations, which referred to insufficient weight and height.

Participants’ understandings of malnutrition went beyond physical references to suggest emotional implications. Participants noted that experiencing emotional distress was related to nutritional status: “this [undernutrition] is due to tension” (Mary). Jon commented that people do not take care of their diet when depressed, and Sakina said that some people do not eat at all and others eat loads when depressed or stressed. Interestingly, various participants viewed junk food as particularly harmful for health and wellbeing: “many people eat fast food. People who are overweight also eat it. They do not take care of their health (…) it [fast food] affects our brain and we have depression” (Rimsha). However, they did not acknowledge experiencing any psychological distress. In the following quote, Qirat’s use of the words “the children”, “they”, and “we” indicates detachment from these experiences. This separation is apparent throughout their narratives and will be analysed in more detail in Theme 3.

*Interviewer: you have written [in your diary] about mental health, so does mental health have anything to do with undernutrition?*

*Qirat: Yes, if there is a fight between the parents, the children also get upset, if they go to school, they do not take interest in studies. It seems to us that they are alright but no, one knows what is going on inside them. I say that it should not be the case because we always have in our minds why this happened today. Then we do not even want to study and then we do not feel hungry. One cannot sleep at night and these thoughts continuously go on in the mind.*

There was a united consensus that weakness was an undesirable consequence of malnutrition. Participants expressed that someone thin, fat or deficient in calcium or iron becomes weak and therefore falls ill and with no appetite: “when people do not take vitamins, they become weak, they also have headaches, and they do not feel hungry. They may have a fever, they will not eat anything” (Mary). Not feeling hungry seemed to be a common concern as it was understood as a sign of illness. Moreover, some participants reported that the weakness produced by being malnourished would impact productivity and physical activity: “under-nutrition… If we don’t consume food, we will become weak. We will not be able to walk again, we will be completely tired. We will not be able to study well” (Mary). It is important to make here a language remark about the word weak (*kamsor*) in Urdu. In the local language, *kamsor* means low weight, but it can also indicate physical weakness, depending on the context. From the participants’ accounts, it appeared that they assumed that someone underweight is inevitably weak.

**Figure 4:**
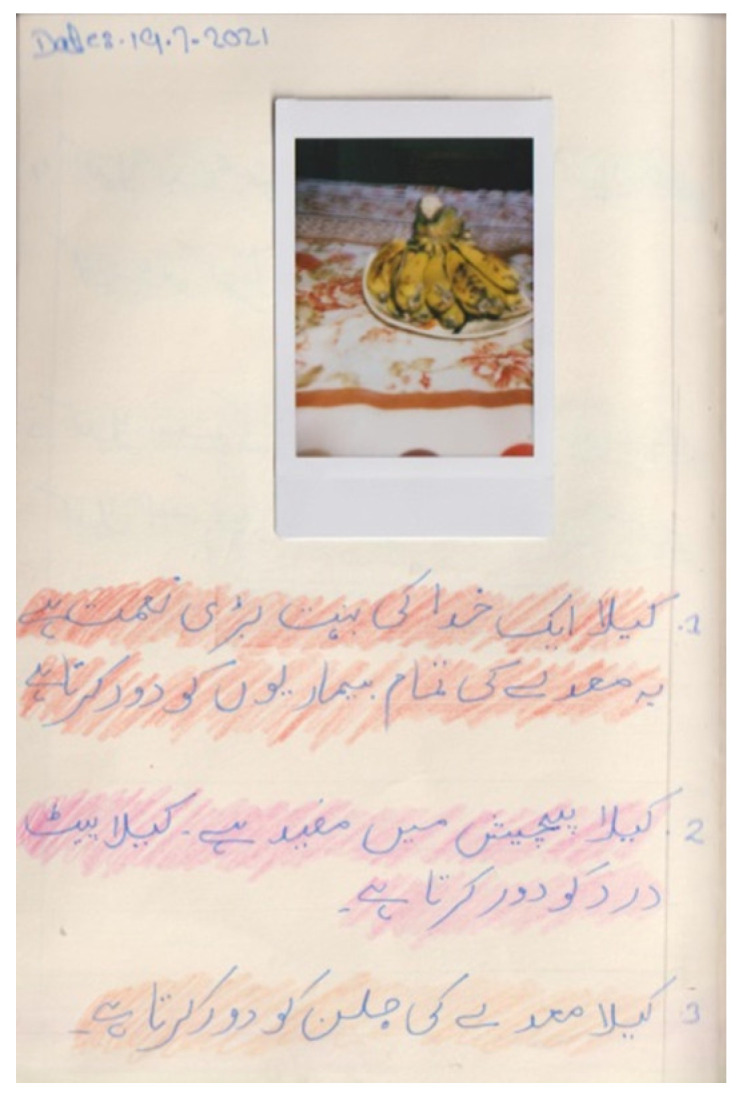
Jannat’s photodiary: “banana is a blessing of God, it can treat all stomach related issues. It is also helpful in dysentery. It cures stomach pain. It treats stomach acidity. [continues on another page, not shown] It cures dyspepsia and nausea. It is also beneficial in heartburn, and it also increases immunity”.

Participants appeared well versed in a wide-ranging of consequences of eating unhealthy food, smoking and not exercising. They wrote in their diaries extensively about unhealthy foods affecting bones, digestive system, eyes, skin, or immune system and causing illnesses such as cardiovascular disease, dental problems, kidney stones, diabetes, or cancer. In the same way, they were also aware of what foods could prevent diseases, to the extent of appointing miraculous qualities; Figure 4. For instance, Sulman wrote in his diary that lemons are good for malaria, and Mahi wrote that rubbing a slice of potato on a burned area removes the burn mark. These are examples of food myths that appeared to be developed from interactions with other people and shaped by local culture.

### 3.2. Theme 2: Who Says What Is Good or Bad?

The knowledge and understanding about food and health seemed to be facilitated by several influencing factors such as family, community, education, friends and media. Compelling the diary involved many participants’ families giving information or even hands-on help. However, participants did not recognise families’ help unless asked, probably because they perceived this project as a ‘school task’. The photodiary instructions given were not specific about this, so participants could turn it into a collective project. For instance, David featured various family members while eating or smoking. Siblings appeared to be the helpers with the making; for example, Sakina’s brother wrote some of the diary pages. By contrast, parents seemed to be the source of knowledge of what is good or bad: “my mother told me about it, that eating tomatoes cure anaemia. (…) When we are not eating the good things that make blood, my mother says to eat strawberries, bananas, tomatoes, chicken, mangoes” (Qirat). Parents prescribed what food must not be eaten. Some participants mentioned that they were not allowed to eat street food as it was considered unhealthy and unhygienically prepared. Yet, other parents let their children eat street food. This behaviour was found very irresponsible as parents were expected to know the adverse effects and prohibit children from eating it. Rimsha commented that parents were the ones to be blamed and responsible for their children’s health since “they [kids] learn whatever they are allowed to eat, if we say this is not good for our health, they will understand and learn”. It became evident that participants’ understandings were influenced by family nutritional knowledge and eating behaviours. In this regard, Sunny’s father could be viewed as a role model as he encouraged healthy food choices at home:

*Sunny: my younger brother always wants to take an outdoor meal, so dad says ‘no, eat a homemade meal, not the outdoor meal, these are good for health’. So my dad also does not take outdoor meal much and prefer to eat homemade food. Dad says ‘eat homemade things. The more you eat homemade things, the better for your health’.*

The harms of eating out versus the benefits of eating homemade food were constantly mentioned during Sunny’s interview. He said that despite the harmfulness and insalubrity of street food, food vendors consciously sell these foods because they have to maintain their business. Sunny also noted that these foods are very appealing and addictive. Other participants similarly noticed that while homemade and unprocessed foods are healthy and germ-free, food from the street makes us sick. These two opposite dietary patterns appeared to be gender-specific: girls eat homemade food, and boys eat out. Mary mentioned that girls are more knowledgeable and more attentive to their diet compared to boys. This may be because girls seemed to learn more from their mothers as they spent more time at home, and mothers appeared to be stricter and more controlling of their daughters. Contrastingly, boys “do not eat that much. They don’t worry about hunger, they just think about going out”, as Mary noted. Qirat felt that boys are not content with the food served at home and go out to eat while girls eat without complaining:

*Qirat: The first thing is that girls eat everything. Even in our house whatever is cooked, we eat quietly. But our elder brothers object that, ‘what it is, what kind of vegetable it is, it is better for me to go out and eat’. All I can say is that boys should eat mostly homemade things, they should know that homemade things are more nutritious. I have two brothers in my house. If they eat lentils during the day, they do not eat the same lentils at night. They order something else from outside at night and say, ‘we did eat this lentil once’. They do not know how much nutrition it will give us if we eat it twice. That is why I would say that boys should eat the same.*

Participants used books from school and information acquired there to write their diaries. Formal education certainly impacted their nutritional knowledge: “we must read. Education shows what is good for us and what is not. (…) And sometimes teachers tell us, this is right for you and this is not” (David). The school was regarded as essential for their personal development and future success in life. It was not only a place to make friends but also a place where food choices needed to be made: “now as the canteen opens, the ones who bring from home are right. Not those who eat canteen stuff, rolls, samosas, pizza, sandwiches, nuggets… This is because they are not good for health. Like buying crust, chocolate, biscuits, not these!” (Sulman). Homemade lunch was viewed as the healthiest option for eating at school as students need good nutrition to “focus on their education” (Mahi).

**Figure 5:**
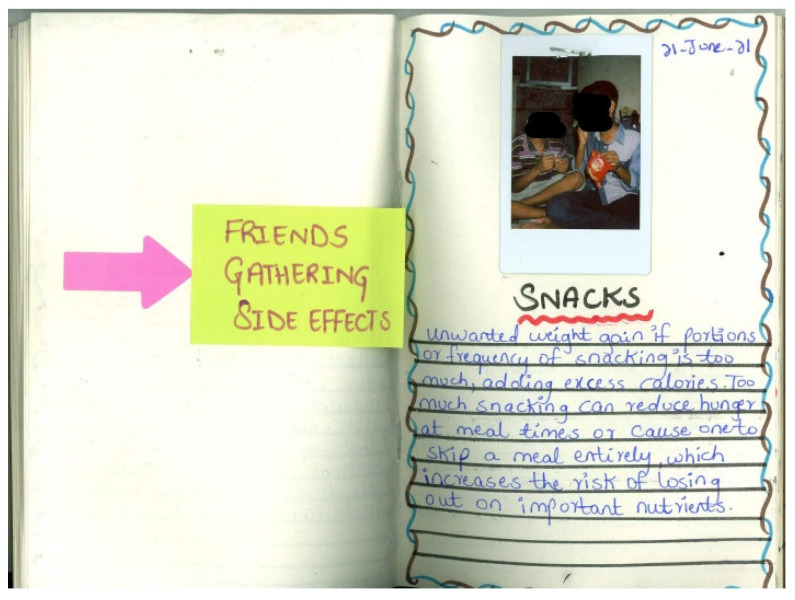
David’s photodiary: Friends gathering side effects. Snacks. Unwanted weight gain if portions or frequency of snacking is too much, adding excess calories. Too much snacking can reduce hunger at mealtimes or cause one to skip a meal entirely, which increases the risk of losing out on important nutrients [written in English].

Friends also appeared to influence participants’ food choices greatly. One participant (Mary) spoke about casual discussions with her friends about what food can be considered good or bad: “he [a friend] said ‘I like pizza very much’. (…) There are green veggies in it. Let’s face it. But there are many things in it that are not good. So the other one said ‘I like this thing very much, we eat meat every day at home’. So we said that is also good”. When Mary and other friends had seen the boy who liked pizza eating “wrong food”, they advised him not to eat it because “it is not good for you” and suggested healthier alternatives instead. Peer pressure was apparent throughout the interviews. Participants mentioned that friends and neighbours insisted on eating junk food and smoking cigarettes, reassuring them that these behaviours do not cause any harm. However, the unwanted consequences of engaging in such unhealthy habits seemed clear; David alluded to gaining weight (Figure 5), and Ali noted addiction. Although having friends was considered important, many participants viewed them as bad influences. There was a general acknowledgement that adolescents become like their friends and cannot be different from them:

*Mahi: If we sit in good company, it will affect our language and everything we do. If we sit with good people who eat good things and meet well, it also affects our health that I will eat what is good thing with them. If we sit with children who talk dirty, eat dirty things, use dirty language, it will affect our health negatively. We try to copy more by looking at our friends. We see them and try to be like them, so we become good, or we become bad. When we sit down with them and get up with them, we will eat what we are eating, so same effect it will have on our health.*

Although this photodiary project was designed not to rely on the internet as limited access was supposed in the participants’ context, at least half of the participants collected information for writing their diaries from the internet. Participants might have used the internet due to the complexity and novelty of the topic or their desire to create good work. As Sakina expressed: “if I make any mistakes, that is not good, isn’t it?”. It can be clearly seen in some participants’ photodiaries that they copied entire pages from the Internet. For instance, Sunny copied the two first paragraphs from the rice entry on Wikipedia [written in English], and David copied a section on the health effects of smoking and tobacco use from the CDC website [written in English]. One participant (Mary) noticed that information from the internet or television ads could be misleading. Nevertheless, the Internet was mainly used to supplement participants’ and their families’ knowledge of (good or bad) food.

*Sakina: I have some previous idea about what are the effects of these outside foods, what are the effects on our body. And if we have to eat outside food, what food we should eat, and how to eat. So I confirmed it from the Internet. I have some personal interests. There was no one with me at that time, so I confirmed it from the Internet.*

Religion also dictated what it was to be consumed. Muslim participants wrote in their diaries that some fruits are a blessing from Allah and that Prophet said that calabash, nigella seeds and dates have healing properties. All participants, regardless of religion, labelled alcohol as very harmful for health, but Ali noticed that a “true Muslim” would never drink or touch it.

### 3.3. Theme 3: Healthy like Us

The majority of participants identified someone around them as overweight, thin or anaemic. Siblings, cousins, school friends were “a bit fat”, “very fat”, “very thin”, “often sick”, “with anaemia”, or “with blood sugar”. However, none of the participants recognised themselves as malnourished, and declared to be healthy and eat properly. This could indicate the coexistence of different nutritional statuses within the same household. Participants were aware that thin or fat bodies were not well regarded in their culture as thin people want to put on weight to “look as good as others” (Sulman), and overweight people “get sick and do not look good” (Zainab). Both thinness and obesity were worrying and avoided due to potential stigmatisation and shame. This probably explains why participants tended to see themselves as healthy and clearly separate themselves from the ‘unhealthy others’. Therefore, it was suggested that people should be “normal”. In the following quote, Sulman explained what he observed from ‘others’ and advised eating a balanced diet:

*Sulman: I have seen that people who don’t go outside and don’t walk a lot, they just eat, they don’t think what they eat, and they just get fat. There are some people who keep fit and even go out for a walk to lose fat. Then, you should not eat too much food, you should eat to a certain extent and you should not eat too little. Whatever you get from nutritious food, you will stay healthy and you will not suffer from any disease.*

**Figure 6:**
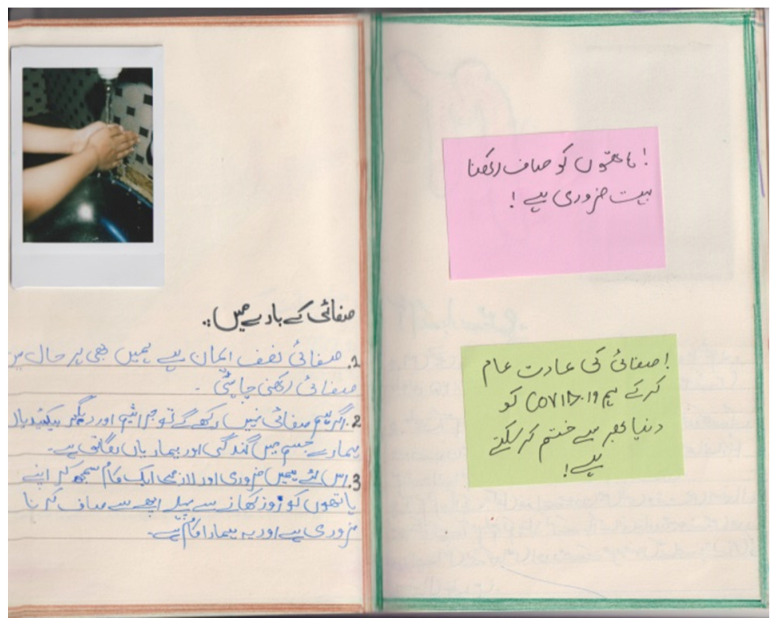
Sulman’s photodiary: Keeping hands clean is very necessary. We can eliminate COVID-19 by making the habit of cleaning part of our daily routine. Cleanliness is half the faith, we should stay clean at all costs. If we don’t keep things clean then these germs and bacteria will affect our bodies and make us ill. That’s why we should consider it important and essential task to wash our hands before eating anything and this is our responsibility.

Throughout the photodiaries and interviews, all participants indicated numerous pieces of advice to be healthy (like them). Alcohol, drugs, tobacco, smokeless tobacco, fried food, non-homemade food, soft drinks, and bad influences should be certainly avoided. They suggested doing exercise and gave guidance on the frequency of consumption of some items; for instance, eating five pieces of fruit and vegetables a day, drinking one glass of milk daily, consuming chicken once per weak maximum, drinking 12 glasses of water per day, and eating an apple, banana, pomegranate or tomato every day. Many participants commented that foods high in micronutrients should be consumed because they delay ageing, allow fast illness recovery, and “prevent our body from becoming weak” (Qirat). Mary used a metaphor to explain that our body has a “syllabus” of micronutrients that we should aim to “maintain”. Participants also gave some hygiene recommendations, such as keeping the kitchen clean, washing fruits and vegetables before consuming, and washing hands before cooking or eating. Some participants highlighted that these recommendations were critical now to stop COVID-19 (Figure 6):

All these recommendations reflect comprehensive health knowledge and understanding of the importance of healthy food habits and lifestyles. Thanks to this knowledge, participants made well-informed decisions; for example, David cut down on chips. Participants mentioned that everyone, but especially adolescents, should know what should be eaten. Sakina commented that adolescents have the ability to learn about nutrition and cooking, so if they are educated on what to eat, they will put the knowledge into practice. Participants themselves and some people close to them actively learned about food and later spread the information. For instance, Ali found that milk works for preventing anaemia and told others about it; and Mahi’s father read about medicine and nutrition because the doctor did not advise Mahi’s ill sister what to eat. However, ‘the unhealthy others’ seemed not to eat mindfully and understand the harms of bad habits. Some participants felt that people were aware of the harmful effects of unhealthy products but consumed them regardless. In any case, participants considered that nutrition and health are in our own hands. As Rimsha said:

*Rimsha: undernutrition or overnutrition is caused by human itself. People should take care of their food. People should not start eating by seeing fast food oily food and not eat. And eat only vegetables, etc.*

People’s responsibility to eat healthily prevailed in participants’ narratives. This is a vivid example of how ‘the others’ are to be blamed for their poor lifestyle choices. Following this responsibility discourse, most participants contemplated merely individually focused health interventions on overcoming the problem of malnutrition in their area. Informing people what to eat seemed to be the most popular measure against malnutrition. Participants suggested nutritional counselling, informing others about the harms and benefits of the foods, displaying ads on the roads, disseminating information by word of mouth, and spreading awareness among adolescents. Jon indicated that not having stress was essential in addition to eating good foods. David commented that he would ask overweight children to exercise and thin children to adopt a healthy lifestyle and take medicines to put on weight. In addition, he would open clinics to treat malnourished people. Some participants viewed a straightforward solution to malnutrition: “tell them to eat and drink, don’t eat these things in excess, eat properly” (Jannat). Furthermore, two participants said that if people do not eat healthily after informing them, “I can’t do anything” (Ali) and “it is their will” (Sunny). Giving food to those in need was considered by several participants to reduce malnutrition in their neighbourhood. Sulman would provide safe and healthy food and filtered water to the poor “so that they will be healthy like us”. Similarly, Sakina would offer food to those around her who don’t have it, but she noticed that people are not so neighbourly nowadays. As we have already noted, there appeared to be a division between the study participants and ‘others’ who needed food or information. The interventions listed by participants did not apply to them and were only for ‘others’ to become “healthy like us”.

Nonetheless, two participants indicated tackling the food environment to support healthier food choices and dietary intake. Rimsha proposed closing restaurants where fast food is sold. Ali suggested, in his own words, that health policy strategies should be evidence-based:

*Ali: I have read so much, and agreed with the chicken fact that it is harmful, it should be consumed once or twice a week. So I will allow chicken sellers accordingly, that is to sell only once or twice a week. The other shops which sell beneficial products will remain open.*

## 4. Discussion

This study demonstrates adolescent sense-making, consciousness and subjective perceptions of malnutrition. Data from photodiaries and interviews generated a novel, thick conceptualisation of adolescent malnutrition and determinants of malnutrition in a slum in Karachi, Pakistan.

In this study, awareness of malnutrition was embedded in participants’ food-centred understandings. Other research in LMIC has documented awareness of childhood obesity and undernutrition from parents’ perspectives [42,43]. Among Indian families, undernutrition was connected to witchcraft as well as feeding habits such as eating age-inappropriate food, not enough food, and not drinking maternal milk [42]. Similarly to our findings, Vietnamese parents related childhood obesity to unhealthy foods, and detrimental health and development [43]. Understandings of obesity causes and consequences were investigated in a sample of children in the UK using focus groups [44]. From an early age, children seem to be aware of a broad scope of negative consequences of obesity [44]. Our participants also recognised numerous consequences of both overnutrition and undernutrition, highlighting weakness as common and severe aftermath. Unlike these children from the UK, our participants manifested physiological perceptions of growth and development. These perceptions rested on the importance of how an individual feels and is viewed (e.g., weak, ill, energetic) as well as bodily dimensions related to weight and height. Although we identified widespread malnutrition awareness among these adolescents living in a slum in Karachi, not all participants were able to articulate a meaning for undernutrition (*ghizayat ki kami*), overnutrition (*ghizayat ki ziyadti*), and vitamin and mineral deficiencies (*haya’teen or madi’niyat ki kami*). Unfamiliarity with these terms could be due to their young age or because they did not identify themselves as thin, overweight or micronutrient deficient. While the Scaling Up Nutrition Civil Society Alliance in Pakistan (SUNCSA-Pak) reported in 2016 that malnutrition awareness spiked in the country [45], we argue that unawareness among adolescents could indicate that this population do not regard malnutrition as a problem in their age group. Further research should investigate the implications of malnutrition unawareness among young populations in LMIC.

Participants’ sense-making around malnutrition constantly showed a pronounced dichotomy between ‘the good’ and ‘the bad’ in regard to food and lifestyle choices. This polarised reasoning may stem from culture, community and household beliefs that are internalised and incorporated into their own narratives. These results are echoed by a recent UK study exploring children’s own perspectives on food choices and factors on eating behaviour [46]. Shared perceptions of diet and health can also arise from cultural gender norms [47,48,49,50]. There was evidence of gendered dietary patterns in our interviews with female participants: boys tended to skip meals or eat out unhealthy food, and girls stayed at home eating homemade food and emulating their mothers. While this study resonates with evidence of the ‘masculine’ behaviour of eating unhealthy foods [49] and the shared perceptions of diet between daughters and mothers [51], it can be argued that the effect of gender stereotype on the adoption of eating habits has to be further studied and contextualised among adolescents living in slums.

The evidence in this study suggests that participants grasped the drivers of malnutrition but did not identify malnutrition as a complex health issue involving structural determinants such as political, geographical and socioeconomic factors. Consistent with qualitative evidence in adolescents, this study identified malnutrition drivers to be sanitation [52,53], exercise [54], families [55], peers [56], wellbeing [57,58], gender [59,60], nutritional knowledge [61], media [56], and most importantly, food [59,62]. Malnutrition appeared to be strongly linked to dietary intake and eating habits in this sample. This could be attributed to several reasons. Firstly, participants perceived that health status (feeling or being seen as weak or strong) depended greatly on what food was consumed. This understanding resembles the ‘you are what you eat’ principle that says “people are believed to take on the properties of the foods they eat” [63]. Secondly, they were aware of the relation between food and body weight, which aligns with previous research [64]. Thirdly, the focus of public health campaigns against malnutrition in their community. Movements such as the SUNCSA-Pak or the WFP operate with a holistic approach targeting all malnutrition determinants as well as encompassing both preventive and curative schemes. On the contrary, other campaigns against malnutrition are run by private companies that present fortified foods as the solution to children’s insufficient eating patterns (e.g., https://runwaypakistan.com/morinaga-nutrition-on-toh-worries-gone/, accessed on 2 March 2022 and https://profit.pakistantoday.com.pk/2021/08/16/millions-of-children-with-malnutrition-need-your-support-sunridge-taqatwar-pakistan/, accessed on 2 March 2022). These products are widely broadcasted on television and online platforms, possibly reinforcing the understanding of ‘malnutrition is all about good or bad food’. Ironically, these specific products were not targeted at people most severely affected by poverty and hence more likely to be micronutrient deficient.

By using photo-elicitation, a global health problem was revealed through the eyes of adolescents living in a slum in Karachi. Despite the challenging nature of the topic and methods, the photodiary created by our participants provided novel insights, facilitated conversation and enriched the interview data. Participants did not provide any meaning or explain their perceptions on malnutrition unless asked in the interview, even though the photodiary instructions indicated “you can write your thoughts, reflections, feelings, and experiences towards undernutrition, micronutrient deficiencies and overnutrition”. Participants demonstrated an appetite for nutritional knowledge. While the photodiary appeared to provide an opportunity for gaining food literacy or reaffirming previous knowledge, the one-to-one interview seemed to evoke reflection on malnutrition meaning and offer nutritional knowledge confirmation. We noted a beneficial and empowering effect of the photodiary, as opposed to the findings from Schwartz and Terry (2017) [38]. Given the attractive and engaging nature of this method, we argue that it can open an opportunity for learning and reflection about health-related topics in adolescent populations.

Participants indicated a clear distinction between the ‘healthy us’ and the ‘unhealthy others’. Although healthy lifestyles and food habits were widely understood in this sample, it was not always evident whether they followed the theory. While food literacy allows healthier and more informed choices [65], knowledge does not necessarily determine behaviour, as food choices during adolescence are more motivated by factors such as food safety, availability and accessibility in LMIC [66,67]. There appeared to be a detachment from their narratives when talking about experiences and health behaviours. Participants expressed that they were healthy but did not exemplify their behaviour using the first person, e.g., “I do” or “I eat”. Instead, participants’ narratives focused on what they observed on the unaware and irresponsible ‘others’ and what the ‘others’ should do, making them solely accountable for their poor lifestyle choices. These narratives align with norms of neoliberal discourse that place responsibility for addressing social problems on the individual. Our findings resonate with previous evidence among Pakistani communities reviling neoliberal narratives of self-critique, self-blame and self-help to resolve their social, well-being and economic issues [68,69,70]. Ultimately, participants’ narratives focused on searching for downstream interventions to address malnutrition in their community, such as providing health information and food donations. These suggested interventions appeared to be targeted only to the ‘others’. Despite the individually focused solutions, a few participants mentioned that improving physical environments could support healthier food choices. It is necessary to better study slum environments to implement effective nutrition programs and upstream interventions to reduce malnutrition in these communities [71].

### Strengths and Limitations

An important strength of this study was its focus on adolescents; qualitative data about adolescents was generated by them and collected from them. This present study appears to be the first qualitative research project to explore perspectives, meanings and awareness of malnutrition among adolescents living in an LMIC and within a slum setting.

The application of photodiaries and interviews in study was useful not only to explore awareness and meanings of malnutrition among adolescents living in a slum in Karachi but also to empower this particular community to learn about nutrition and potentially make healthier food choices. This method or other participatory research action methods using photo-elicitation could allow adolescents in an LMIC to show how they experience important health issues [67,72].

However, some methodological issues need to be considered. Firstly, the research assistant conducting the fieldwork (S.K.Z.Z.) found it challenging to encourage long and elaborate responses in both photodiaries and interviews. Secondly, the researcher interpreting the data (S.E.-Q.) is an outsider who was not present during the fieldwork, limiting the understanding and interpretation of this highly contextual and geographically bounded study. Thirdly, some meaning from the translated interviews and photodiaries might have been lost in backtranslation. However, the constant assistance from the wider research team in the field mitigated this.

Out-of-school adolescents were not included in this study. Since large numbers of out-of-school children and adolescents live in Pakistan (5.5 million) [73], future research should investigate how malnutrition is understood and perceived by these individuals who potentially are at higher risk of malnutrition and adverse health outcomes. Our selection criteria did not include ownership of electronic devices with internet access, minimising selection bias to higher socio-economic groups in the slum. However, the low attendance due to the COVID-19 pandemic and the student’s exams period led to selecting the most reliable students. In addition, our participants had a certain interest and/or understanding of nutrition and health that enabled them to pursue this project more successfully than those students who refused to participate.

Topics such as gendered dietary patterns or individual responsibility discourse were identified but not explored in detail as these were beyond the scope of this particular study. A slum environment has yet to be explored through a feminist and neoliberal governmentality lens to better understand adolescents’ perceptions and meanings of malnutrition.

## 5. Conclusions

This study provides adolescent sense-making around malnutrition and demonstrates malnutrition awareness among 13-16-year-old students living in a slum in Pakistan. In this study, malnutrition was felt as weakness and understood as a lack or excess of food. The findings demonstrated a broad range of factors influencing adolescent malnutrition in this context. The results further suggest that effective interventions for improving adolescent nutritional status need to explore their perspectives and awareness of gender norms and political, geographical and socioeconomic determinants in relation to malnutrition. As evidenced by this study, adolescents have the ability to not only participate in research but also to generate insightful data. To address this global health problem, adolescents should be involved in creating research, solutions and decisions about them.

## Figures and Tables

**Table 1:** Participants characteristics.

Participant’s Name *	Age	Sex	Religion	Recruitment
Jon	13	Male	Christian	Door-to-door
Mary	15	Female	Christian	Door-to-door
David	14	Male	Christian	Door-to-door
Sunny	13	Male	Christian	Door-to-door
Sakina	16	Female	Muslim	Door-to-door
Ali	16	Male	Muslim	School A
Sulman	15	Male	Christian	School A
Qirat	16	Female	Muslim	School A
Zainab	16	Female	Muslim	School A
Ashi	16	Female	Muslim	School A
Rimsha	15	Female	Muslim	School B
Mahi	14	Female	Muslim	School B
Jannat	16	Female	Muslim	School B
Laraib	15	Female	Muslim	School B

* These names are not participants’ real names.

## Data Availability

The participants were told photodiaries and interview data would remain confidential and would not be shared. The first author has full access to the data reported in the manuscript.

## Supplementary Materials

The following supporting information can be downloaded at: https://www.mdpi.com/article/10.3390/nu15010033/s1, Supplementary Material S1: photodiary instructions; Supplementary Material S2: topic guide; Supplementary Material S3: reflexivity [74,75].
