# Supplementary material for: “Good Food Causes Good Effects and Bad Food Causes Bad Effects”: Awareness, Meanings and Perceptions of Malnutrition among Pakistani Adolescents Living in Slums through Photodiaries and Interviews
Source: Nutrients. 2022 Dec 21;15(1):33. doi: 10.3390/nu15010033 (PMC9824795; doi:10.3390/nu15010033)
Supplement: Supplementary file 1 [file nutrients-15-00033-s001.zip › Supplementary Material S1.pdf]

SUPPLEMENTARY MATERIAL 1 – PHOTODIARY INSTRUCTIONS

Now that you know how your new Polaroid camera works… LET'S GET STARTED WITH THE PHOTODIARY!!!

A photo-diary is a record of your day to day life and experiences in which you use both photos and narrative. Entries report both mundane and unusual goings-on, your emotions, thoughts and feelings, your actions and reactions, as well as opinions.

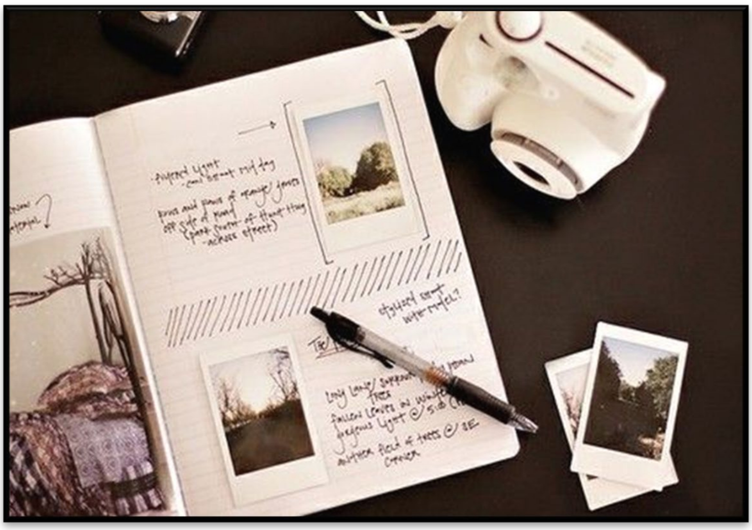

A photodiary could be a fun personal photography project for you: **write and photograph anything that you think is related to undernutrition, micronutrient deficiencies and over-nutrition (malnutrition) in the notebook** provided and coloured as much as you want with the pens.

How you create this photodiary is completely up to you. Here you have the procedure usually followed to produce a photodiary. However, feel free to follow these steps.

- Write down the date of the entry.

- Firstly, think about what is the issue that you want to talk about. For this photo-diary, **<u>we are asking you to focus on these topics: undernutrition, micronutrient deficiencies and overnutrition</u>**.
- Photograph it and paste the photo in the notebook.
- Write some paragraphs about the photo: what do you see? Why it is important for you? How this is related to undernutrition, micronutrient deficiencies or over-nutrition? What are your feelings towards it?
- You can write your thoughts, reflections, feelings, and experiences towards undernutrition, micronutrient deficiencies and over-nutrition.
- Use some colours pens to draw something related with your ideas or highlight your points.

We want you to create your photodiary as you like, but if you are looking for some inspiration, here you have some tips:

- You can write, draw, stick photograph, stick newspaper, write stories, magazine images, Rapper or anything related to undernutrition, micronutrient deficiencies or over-nutrition.
- You can take inspiration from TV, Radio, dramas, movies, advertisements and daily conversation etc.

Some things to bear in mind:

- Whenever you take anybody else's picture you will need to ask for their consent.
- When making your photodiary, feel free to be as exhaustive and creative as you like.
- There is no wrong or right answer; everything you write, think, answer and ask is right!

<u>Your questions answered:</u>

How frequently should I make entries in my photodiary?

As frequently as you like. However, we would like you to write a minimum of 7 entries.

How much time do I have to produce my photodiary?

You have two weeks. After these two weeks, you should bring your photo-diary at school and the researcher will collect it. Once she has scanned it, she will give it back to you so you can keep it for yourself.

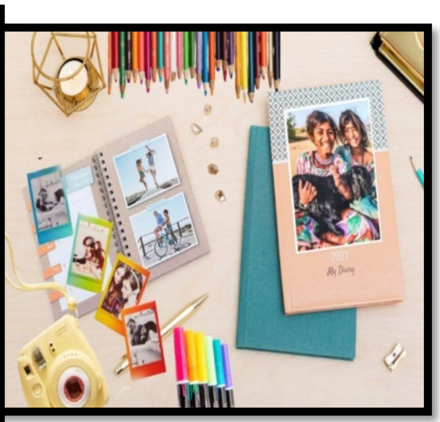

We hope that you enjoy the creation of this photodiary and thank you very much for participating.

Dear participant,

Thank you so much for participating and creating this photodiary. As you know, we asked you to write and photograph anything that you think is related to undernutrition, micronutrient deficiencies and overnutrition in the notebook. Now, we want to ask you another question.

- What do you think that <u>worsens and improves</u> **undernutrition**? Why do you think so?
- What do you think that <u>worsens and improves</u> **micronutrient deficiencies**? Why do you think so?
- What do you think that <u>worsens and improves</u> **overnutrition**? Why do you think so?

We know that these questions are hard to answer so here you go some ideas of what can worsen or improve undernutrition, micronutrient deficiencies, and overnutrition:

**Diet**	**Washing habits, hygiene**	**Mental issues**
**Smoking**	**Alcohol use**	**Family**
**Peer support and friendships**	**Physical activity and sedentary behaviour**	**Education, schooling**
**Other diseases**	**Cultural norms**	**Food environment**
**Gender norms**		

Feel free to add or remove words from this list. You can choose which ones you want to talk about.

Please, write and photograph about these question in your diary following the same instructions that we gave you at the beginning of the study.

Bear in mind that we will collect your diary in a week from now.

We hope you enjoy creating this photodiary!
