# Supplementary material for: “Good Food Causes Good Effects and Bad Food Causes Bad Effects”: Awareness, Meanings and Perceptions of Malnutrition among Pakistani Adolescents Living in Slums through Photodiaries and Interviews
Source: Nutrients. 2022 Dec 21;15(1):33. doi: 10.3390/nu15010033 (PMC9824795; doi:10.3390/nu15010033)
Supplement: Supplementary file 1 [file nutrients-15-00033-s001.zip › Supplementary Material S2.pdf]

SUPPLEMENTARY MATERIAL 2 – TOPIC GUIDE

Tell me a bit about yourself (icebreaker questions).

- Can you talk me though a typical day? What do you like to do during the weekends? Leisure activities, school...

Tell me how the experience of writing a photo-diary was

- How was the experience of taking pictures? Did you like it? What did you like about this experience? Did you have any problem while taking pictures? Was the camera working well?

Now I am going to ask you some questions about your photodiary, so it would be great if you have it in front of you.

- 3th June page – side effect of tobacco: Why do you think that smoking is bad? How this affects your dad/granddad? How do you feel about it?
- How did you know about all this side effects? Did you look it up on the Internet? Why did you go to the Internet? Did anyone help you with the photo-diary and looking up things online? I see that you photographed your friends and family, how did you come up with the idea? Why did you choose to photograph people too?
- 8th June – benefits of milk: how did you learnt about these micronutrients?
- 9th June – side effect of bread & benefits: I am very interested to know what “antinutrients” are. Apart from bread, what food you know that is often enriched with “extra nutrients”? What extra nutrients are those? Where did you learn about enriched foods (media, school…)?
- 11th June – side effect of broast: where did you get this information from? Who are these scientists? you mentioned that friend foods can lead to obesity, can you name some other foods that can produce obesity?
- 21st June - Wine : how do you think that there is a relation between alcohol and malnutrition?
- 22nd June – hand wash: what happens if you don’t wash your hands before eating food or after the toilet? How do you think that this has an impact on malnutrition?

I would like to know about undernutrition and overnutrition in your community, especially among adolescents.

<u>Meaning and awareness</u>:

- What do you understand by undernutrition? And overnutrition? (if the participant does know what undernutrition / overnutrition means, the research assistant will explain it to them so the participant is able to answer the rest of the questions) how do you know this?

<u>Experience</u>:

- How would you describe your experience with undernutrition? And with overnutrition? How does undernutrition affect your day to day? How does

undernutrition relate to your life? How does undernutrition affect you? How do you feel about it?

- Do you think that undernutrition and overnutrition are somehow related?

Context:

- Where there is under and over? In what situation? (Does it happen in adolescents living in Pakistan? And in your area?) What are your thoughts on undernutrition in your school and community? And on overnutrition?

Determinants:

- To your understating, why do you think that undernutrition happens? (if needed, prompt factors / behaviours associated with undernutrition such as sanitation, gender, setting, age)
- In the second week instructions, there is a table with things that may worsen or improve malnutrition. In your experience, can you name what worsens undernutrition? And what improves undernutrition? What do you think that worsens and improves overnutrition? Ask for more factors if they only give one or two examples.
- What do you think they could do to improve malnutrition in adolescents? And in your community?

I think that's basically everything I had to ask you. Have you got anything else you would like to say or any kind of final thoughts or anything you would like to follow up that I haven't asked you
