# Supplementary material for: “Good Food Causes Good Effects and Bad Food Causes Bad Effects”: Awareness, Meanings and Perceptions of Malnutrition among Pakistani Adolescents Living in Slums through Photodiaries and Interviews
Source: Nutrients. 2022 Dec 21;15(1):33. doi: 10.3390/nu15010033 (PMC9824795; doi:10.3390/nu15010033)
Supplement: Supplementary file 1 [file nutrients-15-00033-s001.zip › Supplementary Material S3.pdf]

Supplementary material 3 – reflexivity

Two main individuals shaped the making of this cross-cultural, cross-language research project: Syeda Kisa Zehra Zaidi (as the research assistant) and Sara Estecha Querol (as the researcher). The following critical reflection was written by SEQ.

As the researcher, I am aware that my persona, research goals and expectations, previous experiences, and assumptions would (and did) influence the research process. In order to establish rigour and transparency, a critical reflection about my role in the study, a personal statement of the research assistant, and a contextual consideration of our relationship are provided.

Starting with my persona, I am a Spanish citizen born in 1991 (she/her). I have a background in Nutrition and Public Health, and my research interests include adolescent health, global health nutrition, health policy agenda and participatory action research methods. During my PhD program (where this project is located), I planned to develop my research skills in mixed-methods and critical qualitative research. One of my PhD goals (and expectations) was to work together with adolescents living in a slum to understand adolescent malnutrition and trigger some social change rather than merely collecting data to explore adolescent malnutrition and construct an interpretation (my interpretation vs. our [their+my] interpretation). However, the COVID-19 pandemic started after designing the PhD project but before carrying out the qualitative study. This meant loads of frustration, removing the participatory aspect and consequently re-planning the project. As a visual learner, I am inclined to visual methods, especially when it comes to a cross-cultural, cross-language research project because I believe that images are a powerful mean of communication and have the potential to minimise the language barrier ("a picture is worth a thousand words"). Hence, it is very likely that my interest in visual and participatory methods influenced the selection of the qualitative research method.

Indeed, I aimed to minimise the language barrier to some extent by using images and working with a research assistant as a translator. However, I did not aim to minimise cultural differences. Instead, I tried to acquire cultural sensitivity and competence by reading books, research papers, asking and actively listening to Kisa, and keenly observing the dynamics within the research team to learn about the culture, religion, history and politics of the country and the particular study site. Immersing in a culture (i.e. long stay within the group) may provide a researcher with an in-depth and accurate understanding of a linguistically and

culturally distant community (Liamputtong, 2010). However, a long stay in Karachi was not an option at that time.

The role of research assistants or interpreters has been hardly investigated as usually, the researcher claims full ownership of the research process and the findings (Stevano & Deane, 2017). Unquestionably, the research assistant had a crucial role in this project which came with great responsibility towards the research process. From my perspective, it was crucial that authority and power asymmetries rooted in both the employment relationship and our foundational knowledge were reviewed. Only then Kisa could also own the project so we (Kisa and I) could ensure the success of *our* project (Kisa referred to this project with the possessive pronoun “our”).

Providing quality training on the study’s aims and methods to Kisa as well as staying closely in contact with her was critical and had a very positive impact on the research process and quality of the outcomes. While she provided me with local and cultural knowledge, I taught her from qualitative theoretical foundations and methodology to practicalities on how to navigate academia. The contextual intersecting relationship between Kisa and I went beyond a hierarchic professional relationship where I was a mentor to her and she was my trusted eyes, mouth and ears in the field. Kisa was the only person through whom I could live the experience of undertaking this study, so I asked her to document her trips to the field in great detail for not only quality data collection purpuses but also for my own learning. Kisa also wrote a self-reflection on her performance during the interviews so she could improve her interviewing skills next time.

Reflecting on how Kisa and I shaped the project, I realised that this reflection not only needs to come from my lens but also from Kisa’s perspective, so I asked her to share some lines with her views.

“I am Syeda Kisa Zehra Zaidi; I acted as a research assistant in this project. I am a Public Health Nutritionist by profession and currently pursuing my MS degree in Public Health. I am a citizen of Karachi (Pakistan), where this study was carried out.

Initially, I was ambiguous regarding the execution of cross-cultural research. I had an assumption that it might be challenging for Sara to relate with the kids from a slum in a developing country. But as we progressed, we found different ways to explain and understand these cultural differences. My socio-demographic background supported me a lot throughout the research. My education and experience served as a bridge between Sara and the participants, and these also helped me absorb the photodiary method and research topic. The

food myths that participants mentioned during the interview or within the photo diary, I was aware of them all. However, I noticed a significant gap between Sara and the participant's culture, a lot of these foods myths were novel findings for Sara.

Like any other researchers, we had also encountered plenty of issues: COVID lockdowns, alternate schooling days, travel restrictions, and connectivity issues. As per my assumption, this study might have been executed way more smoothly in a pre-COVID era in the presence of Sara. I absolutely enjoyed the participants' excitement for participating in this study, capturing pictures, designing diaries, and giving interviews, which, unfortunately, Sara missed witnessing. Being a female, communicating with parents and recruiting the participants (especially females) was more feasible due to parents' safety concerns. As observed, Pakistani parents trust females more in a scenario that involves interviews and taking pictures with a Polaroid camera. The lesser age gap between the participants and me made it easier for me to explain the study."

The fact that the age gap between Kisa and participants was not more than ten years might have made the adolescents feel comfortable and not see her as a researcher 'examining' them. Her cultural similarity and familiarity (vs mine) with participants, their families and the school staff facilitated trust and rapport. At this point, it may be very apparent the duality of insider versus outsider in this qualitative project. Kisa, as the local researcher, was linguistically and culturally close to the participants, while I was distant from all (Kisa and the research participants). From her insider perspective, it is important mentioning that Kisa had no previous contact with the study site community. From my outsider perspective, I was an external researcher who was not present in the data collection. I am very aware of my condition as a Western white woman leading a cross-cultural research project, and I question if my writing legitimates and interprets the people whom I researched. It is obvious that I shaped the making of this research study but tried that these characteristics did not influence the research process, participants or research assistant. On the bright side, being an outsider has "its own usefulness, such as providing different perspectives on cultural and community norms, and asking questions that require more detailed explanations" (Coloma, 2008 cited in Liamputtong, 2010). Additionally, being a remote outsider could be advantageous too, as the flow of the recruitment and data collection was not being disturbed by my whiteness and womanness.

References

Liamputtong, P. (2010) *Performing Qualitative Cross-Cultural Research*, Cambridge: Cambridge University Press.

Stevano, S. and Deane, K. (2017) The Role of Research Assistants in Qualitative and Cross-Cultural Social Science Research, In P., L. (ed) *Handbook of Research Methods in Health Social Sciences.* , Singapur: Springer.
